# Comparison of Relation between Attention Deficit Hyperactivity Disorder in Children with and without Simple Febrile Seizure Admitted in Arak Central Iran

**Published:** 2016

**Authors:** Bahman SALEHI, Parsa YOUSEFICHAIJAN, Smira SAFI ARIAN, Somaieh EBRAHIMI, Mahdyieh NAZIRI

**Affiliations:** 1 Psychiatric Department, Arak University of Medical Sciences, Arak, Iran; 2 Pediatric Nephrology Department, Nephrology Department, Arak University of Medical Sciences, Arak, Iran; 3 General Practitioner, Arak University of Medical Sciences, Arak, Iran; 4 Psychology Department, Islamic Azad University of Arak, Arak, Iran; 5 Base Sciences Department, Clinical Research Office of Amir almomenin hospital, Arak University of Medical Sciences, Arak, Iran

**Keywords:** Children, Febrile seizure, ADHD

## Abstract

**Objective:**

Febrile seizure is one of the most prevalent childhood convulsions with the most common age of onset at 14-18 mo old. Fever decreases the brain threshold for seizure. Attention Deficit Hyperactivity Disorder (ADHD) is also a neurologic-behavioral problem defined by attention deficit and hyperactivity according to DSM-IV criteria in which the child must have these signs in two different environments. There is controversy on the possible relation between febrile seizure and ADHD; while some studies approve a strong relation, some exclude any relation and some attribute ADHD to the side effects of other reasons.

**Materials & Methods:**

This descriptive-analytic study enrolled all children of 3-12 yr old with febrile seizure (according to Nelson Pediatrics Textbook diagnosed by the pediatrician in charge) referring to Amir Kabir Hospital, Arak, central Iran in 2010-2011.

Overall, 103 of them with no corporeal or psychological disorder (like depression, anxiety, schizophrenia and other CNS maternal disease) were compared to 103 children of the same age and gender admitted due to disease other than febrile seizure utilizing DSM IV criteria for ADHD. Data were analyzed using SPSS version 18.

**Results:**

The hyperactivity disorder in the control and case group was 34.3% and 16.7%, respectively, denoted a significant relation between simple febrile seizure and hyperactivity.

**Conclusion:**

Hyperactivity has a significant relation with febrile seizure in male gender, making further investigation in these children prudent for early diagnosis and management.

## Introduction

With the prevalence of 2% to 5%, febrile seizure is the most common childhood seizure. The most common age at onset is 14-18 months. Incidence of febrile seizure before 9 months and after 5 yr of age suggests underlying neurological abnormality.

Febrile seizure is inherently a benign process (1). Recurrence of febrile seizure is about 30%, but epilepsy has only been reported in 2% of cases. Fever leads to seizure by reducing brain threshold. Positive familial background has been reported in 25% to 40% of cases; it also has genetic background as Autosomal Dominant (AD) (1).

Febrile seizure is when the focus of local infection cannot be detected, and is divided into two groups of simple and complex seizures. A simple febrile seizure is a primary generalized, usually tonic-clonic, attack associated with fever, lasting for a maximum of 15 min, and not recurrent within a 24-h period. “A complex febrile seizure is; more prolonged (>15 min); is focal,; and/or recurs within 24 h” (2). Generally, prognosis of febrile seizure in children is excellent. Over the past 20 yr, treatment of children with febrile seizure has dramatically changed. However, controversies exist among pediatricians regarding treatment of this common childhood problem. 

Attention Deficit/Hyperactivity Disorder (AD/HD) are among the most controversial childhood disorders, and lead to referrals to child psychiatrists and consultants more than any other single disorder. This disorder profoundly affects lives of thousands of children and their families, and has improper developmental symptoms (attention deficit, hyperactivity, and impulsivity). 

Various possible causes have been proposed for AD/ HD, including genetics, organic, environmental, or a combination of these (3). Studies have been conducted on the relationship between simple febrile seizure and AD/HD, with contradictory results. Some emphasize a strong relationship between the two and some report no relationship at all (4-10). 

Children with first seizures had greater behavioral disorders over the 3-yr follow-up (4). This 3-yr long study of children with first seizures aimed to determine the basic difference in behavioral disorders between children with and without seizures and risk factors of behavioral disorders over 3 yr following seizure. Risk factors included age, gender, and neuropsychological factors such as IQ, seizure, and familial factors. Key risk factors included low education of caregivers, reduced family support for children’s independence, and low confidence of parents in children’s capabilities, which increase the risk of behavioral disorders in children (4). In another study, children with seizures had greater internal and external behavioral disorders compared to healthy children or children with other chronic diseases (5). The risk of neuropsychological disorder, sleep disorders were greater in children with IQ>70 and first While there was higher performance scores and showed no tendency toward impulsivity (6-9). 

In a study, AD/HD diagnostic interview program based on DSM-IV, showed that incidence of AD/HD disorder in children with first seizure was 2.5 times greater than in control group. This was significantly associated with attention deficit disorder rather than hyperactivity or combination type of these disorders (10). Besides, early seizure was associated with behavioral and cognitive disorders, even in the absence of structural brain damage, furthermore, these disorders were dependent on age at onset of seizure (less intense behavioral disorders at younger age) and intensity of seizure attacks, but not on causes of seizure or electrically and chemically induced seizures (11). Hence, children with a history of seizure showed greater levels of behavioral disorders, attention deficit disorders and somatic complaints (12). 

Behavioral disorders were 4.7 times more in children with seizure and 3 times more in children with cardiac problems. (13-15).

The aim of this study was providing a better treatment and care for patients with simple febrile seizure through identifying its conditions. 

## Materials & Methods

In this descriptive-analytical study, the study population consisted of all 3-12 yr-old children, attending Amir- Kabir Hospital in Arak during 2011 and 2012, with diagnosis of simple febrile seizure (according to above definition). According to consultation with statisticians and simple random sampling, sample size consisted of 103 3-12 yr-old children with simple febrile seizure (as diagnosed by the pediatrician at Amir-Kabir Hospital), with no history of physical or mental diseases such as depression, anxiety, schizophrenia, or congenital diseases (case group), and 103 children of similar age and gender admitted to the hospital without simple febrile seizure (control group). Patients’ history (control and case groups), checklist prepared from Kaplan book based on DSM-IV, and demographic details of all patients (case and control groups) were completed by project executers and thesis intern. Necessary coordination was made with project psychiatrist to ensure patients met AD/HD criteria according to the checklist. Patients not qualifyed for febrile seizure definition such as children with focal seizures, frequent seizures (more than once) in 24 h, duration of seizure longer than 15 min, and possible CNS diseases such as meningitis, encephalitis, and possible epilepsy (given previous frequent seizures) were excluded from the study. 

Parents or guardians of patients were able to withdraw their children from the study at any stage. They provided us an informed consent before the study. Statistical tables and figures as well as tests for comparison of mean in the independent groups and comparison of ratios were used. Collected data were analyzed with SPSS software, and t-test was used to compare control and case groups. P<0.05 was determined significant.

## Results

Table 1 presents distribution of frequency and relative frequency of attention deficit/hyperactivity, impulsivity disorders, and combination of these in study groups according to gender. None of male children with simple febrile seizure and 6.1% of the control group had attention deficit, 34.3% of male children with simple febrile seizure and 16.7% from the control group had hyperactivity/impulsivity disorder, and 34.3% of male children with simple febrile seizure and 18.2% from the control group had combined disorders, and that prevalence of these disorders was not the same in the two groups. Conversely, none of female children with simple febrile seizure and 2.7% of the control group had attention deficit, 23.1% of female children with simple febrile seizure and 21.6% from the control group had hyperactivity/impulsivity disorder, and 21.3% of female children with simple febrile seizure and 21.6% from the control group had combined disorders, and that prevalence of these disorders was the same in the two groups. Table 2 shows a significant difference in mean age of mothers in the two groups. 

According to results presented in Table 3, the frequency of consanguineous marriages was 26.2% in parents of children with simple febrile seizure, and 9.7% in the control group (P=0.03), thus, consanguineous marriages are not equally distributed in the two groups. Based on the completed questionnaire, none of the patients had any family history of psychiatric diseases in their parents or siblings. 

## Discussion

According to results, hyperactivity was more prevalent in male children with simple febrile seizure compared to children without, which suggests a relationship between simple febrile seizure and hyperactivity in boys. However, no significant difference was found in the prevalence of hyperactivity between female children with simple febrile seizure and control group. Attention deficit showed no increase in any of the male and female children with or without simple febrile seizures. Hyperactivity/impulsivity showed greater increase in male children with simple febrile seizure compared to the control group, while in female children the increase was not significant. Moreover, the results indicate much higher prevalence of simple febrile seizure among children with a history of consanguineous marriages compared to children without such a history. These results concur with another study, in which children with first seizure showed greater behavioral disorders over 3-year follow-up (4). Furthermore, in children with first seizure, greater internal and external behavioral disorders compared to healthy children or children with other chronic diseases was noticed. Neuropsychological and behavioral functions, especially practical function predict behavioral outcomes 3 years after the onset of seizure. The present study showed that hyperactivity disorder was greater in boys with simple febrile seizure, which increases the risk of hyperactivity/ attention deficit in them, which is in line with the above study (5). These children were more exposed to the risk of neuropsychological disorder, which is in line with the present study. The present study showed that hyperactivity risk was greater in boys with simple febrile seizure, which concurs with the conducted study (6). A study showed greater sleep disorders (including: timing of sleep and sleepiness during daytime) in children with seizures (7). In the present study, hyperactivity disorder was greater in male children with simple febrile seizure, which agrees with the above study. After 6-year followup, febrile seizure group had no greater behavioral disorders than the control group, whereas in the present study, greater risk of hyperactivity (and hyperactivity disorders) was shown in boys with simple febrile seizure, but no increased risk of attention deficit was observed, which disagrees with the above study (8, 9). 

**Table 1:** Frequency and Percentile Distribution according to Gender and ADHD Subtypes (Attention Deficit, Hyperactivity - Impulsivity, and Combined) in Groups with and without Simple Febrile Seizure admitted in Arakamir-Kabir Hospital on 2010-2011

**Disorders**			**Attention Deficit**							**Hyperactivity** ** -** ** Impulsivity**									**Combined**									
**Groups**			**Case**			**Control**				**Case**						**Control**			**Case**				**Control**					
**Sex**			**Frequency**	**Percentile**		**Frequency**			**Percentile**	**Frequency**				**Percentile**		**Frequency**		**Percentile**	**Frequency**	**Percentile**			**Frequency**					**Percentile**
**Female**		**Yes**	0		0		1	7.2		9	1.23				8		6.21		9	1.23				8	6.21			
		**No**	39		100		36	3.9		30	9.76				29		4.78		30	9.76				29	4.78			
**Total**			39		100		37	37		39	100				37		100		39	100				37	100			
**Pvalue**			P=0.4							P= 0.05									P=0.05									
**Male**		**Yes**	0		0		4	1.6		22	3.34				11		7.16		22	3.34				12	2.18			
		**No**	64		100		66	100		42	6.65				55		3.83		42	6.65				54	8.81			
**Total**			64		100		66	100		64	100				66		100		64	100				66	100			
			P=0.05							P=0.017									P=0.046									
**Female** **and Male**	**Yes**		0		0		5	4.9		31		30.1			19		18.4		31		30.1			20		19.4		
	**No**		103		100		98	95.1		72		69.9			84		81.6		72		69.9			83		80.6		
**Total**			103		100		103	100		103			100		103		100		103			103		103			100	
**P value**			P=0.059							P=0.07									P=0.01									

**Table 2:** Comparison of Age, Maternal Age, Birth Weight between Groups with and without Simple Febrile Seizure in children Admitted in Arak Amir-Kabir Hospital On 2010-2011

**Statistics**		**Frequency**	**Mean**	**Standard-Deviation**	***P*** **-value**
**Group**					
**Age**	**Case**	103	61.55	22.589	0.951*
	**Control**	103	61.75	22.559	
**Maternal Age**	**Case**	103	23.81	3.367	0.045**
	**Control**	103	24.90	4.365	
**Birth Wt.**	**Case**	103	3052.72	386.382	0.847***
	**Control**	103	3039.32	536.129	

**Table 3:** Frequency and Percentile Distribution of Family History of Attention Deficit and Hyperactivity in Both Groups with And without Simple Febrile Seizure in Children Admitted in Arak Amir-Kabir Hospital on 2010-2011.

**Statistics**			**Case**	**Control**	***P*** **-value**
**Group**					
**Family History**	**Yes**	**Frequency**	**1**	**0**	**0.05** *
		**Percentile**	**1**	**0**	
	**No**	**Frequency**	**102**	**103**	
		**Percentile**	**99**	**55**	
**Total**					
**Familial Marriage**	**Yes**	**Frequency**	**27**	**10**	**0.5** **
		**Percentile**	**26.2**	**9.7**	
	**No**	**Frequency**	**76**	**93**	
		**Percentile**	**73.8**	**90.3**	
**Total**					
**Total**	**Case**				**0.837** ***
	**Control**				

Children with first seizures (3-16 yr-old), hyperactivity/ attention deficit disorder in these children was 2.5 times more compared to the control group. This was significantly associated with attention deficit, but not with hyperactivity or combined form, and seizure, cause of seizure or gender played no role in results. In the present study, the risk of hyperactivity/attention deficit disorder was greater in boys with simple febrile seizures, and gender had no effect on febrile seizure, which agrees with the above study (10). They had greater intellectual problems. Moreover, children with previous seizures scored higher marks in behavioral disorders, somatic complaints, depression, anxiety, intellectual problems and attention disorder. In the present study, hyperactivity in boys with simple febrile seizure showed higher increase compared to the control group, which is in line with the above study (11). The effect of behavioral and cognitive disorders due to seizure on brain showed that early seizure was associated with behavioral and cognitive disorders, even in the absence of structural brain damage. These disorders depended upon age at the onset of seizure (less intense behavioral disorders at younger age), intensity and seizure attacks, and not upon cause of seizure or electrically and chemically induced seizures. In our study, hyperactivity showed greater increase in boys with simple febrile seizures compared to control group, which agrees with the above study (12). Higher levels of behavioral disorders were observed among children with previous seizure attacks (13). Therefore, children with a history of seizure showed greater levels of behavioral disorder, attention deficit disorder and somatic complaints, which agrees with the above study (13). 

According to our results, there was a significant relationship between male hyperactivity and simple febrile seizure. Thus, preventive diagnosis in terms of likelihood of hyperactivity, and early and preventive actions should be performed in male children with simple febrile seizure.

**In Conclusion, **the data showed that hyperactivity has a significant relation with febrile seizure in male gender, making further investigation in these children prudent for early diagnosis and management. 

## Author Contribution

Yousefichaijan P and Salehi B planned and carried out the study. Edited the paper and participated in the follow-up. 

Ebrahimi S and Safi Arian S performed the clinical testing of the patients participating in the study. 

Naziri M performed the statistical analysis and helped to draft the manuscript.

All authors agreed to be accountable for all aspects of the work in ensuring that questions related to the accuracy or integrity of any part of the work are appropriately investigated and resolved. 
